# Supplementary material for: Asynchronous magnetic resonance elastography: Shear wave speed reconstruction using noise correlation of incoherent waves
Source: Magn Reson Med. 2022 Oct 27;89(3):990–1001. doi: 10.1002/mrm.29502 (PMC9792433; doi:10.1002/mrm.29502)
Supplement: Supplementary file 1 — DATA S1 MATLAB code used to generate the numerical simulations of Figure 1 [file MRM-89-990-s002.zip › k-Wave/helpfiles/interpCartData.html]

interpCartData :: Functions (k-Wave)


# interpCartData

Interpolate data from a Cartesian to a binary sensor mask.

## Syntax

```
binary_sensor_data = interpCartData(kgrid, cart_sensor_data, cart_sensor_mask, binary_sensor_mask)
binary_sensor_data = interpCartData(kgrid, cart_sensor_data, cart_sensor_mask, binary_sensor_mask, interp)
```

## Description

`interpCartData` takes a matrix of time-series data recorded over a set of Cartesian sensor points given by `cart_sensor_mask` and computes the equivalent time-series at each sensor position on the binary sensor mask `binary_sensor_mask` using interpolation. The properties of `binary_sensor_mask` are defined by the k-Wave grid object `kgrid`. Two and three dimensional data are supported.

## Inputs

|  |  |
| --- | --- |
| `kgrid` | k-Wave grid object returned by `kWaveGrid` |
| `cart_sensor_data` | original sensor data measured over `cart_sensor_mask` indexed as `cart_sensor_data(sensor position, time)` |
| `cart_sensor_mask` | Cartesian sensor mask over which `cart_sensor_data` is measured |
| `binary_sensor_mask` | binary sensor mask at which equivalent time-series are computed via interpolation |

## Optional Inputs

|  |  |
| --- | --- |
| `interp` | interpolation mode used to compute the time-series, both `'nearest'` and `'linear'` (two-point) modes are supported (default = `'nearest'`) |

## Outputs

|  |  |
| --- | --- |
| `binary_sensor_data` | array of time-series corresponding to the sensor positions given by `binary_sensor_mask` |

## Examples

- 2D Time Reversal For A Circular Sensor
- 3D Time Reversal For A Spherical Sensor
- Image Reconstruction With Compensation For Acoustic Absorption

## See Also

`cart2grid`, `kspaceFirstOrder2D`, `kspaceFirstOrder3D`, `kWaveGrid`
